# Exercise Capacity, Iron Status, Body Composition, and Mediterranean Diet in Patients with Chronic Heart Failure

**DOI:** 10.3390/nu15010036

**Published:** 2022-12-21

**Authors:** Pauline Bayerle, Sebastian Beyer, Uwe Tegtbur, Momme Kück, John Adel, Stefan Kwast, Christoph Pökel, Arno Kerling, Martin Busse

**Affiliations:** 1 Department of Rehabilitation and Sports Medicine, Hannover Medical School, Carl–Neuberg–Str. 1, 30625 Hannover, Germany; 2 Department of Cardiology and Angiology, Hannover Medical School, 30625 Hannover, Germany; 3 Institute of Sports Medicine and Prevention, University of Leipzig, 04109 Leipzig, Germany

**Keywords:** chronic heart failure, nutrition, exercise capacity, body composition, dietary behavior, mediterranean diet, iron status, ferritin

## Abstract

In addition to drug therapy, lifestyle modification, including physical activity, and nutrition management are an integral part of current guidelines for patients with chronic heart failure (CHF). However, evidence on which clinical parameters are most influenced by nutritional behaviour, exercise capacity, or iron status is scarce. For a multicenter intervention study, we included participants with diagnosed CHF (*n* = 165) as well as participants with elevated NT-proBNP values and risk factors for CHF (*n* = 74). Cardiorespiratory fitness was tested with a bicycle test, and adherence to the Mediterranean diet (MedDiet) was assessed with the MDS questionnaire. Our data strengthened previous results confirming that the higher a person’s adherence to MedDiet, the higher the cardiorespiratory fitness and the lower the body fat. Furthermore, our results showed that anemia in patients with CHF has an impact in terms of cardiorespiratory fitness, and functional outcomes by questionnaire. Since our data revealed gaps in iron supply (37.9% with iron deficiency), malnutrition (only 7.8% with high adherence to MedDiet), and both symptomatic and non-symptomatic study participants failed to meet reference values for physical performance, we encourage the enforcement of the guidelines in the treatment of CHF more strongly.

## 1. Background

Chronic heart failure (CHF) is one of the most common diseases with a prevalence of 1–2% in adults in developed countries, disproportionately affecting the elderly [1,2]. As studies usually include diagnosed CHF cases, the true prevalence is likely to be higher [3]. The causes of its development are manifold, in western civilizations up to 90% of cases are due to cardiovascular comorbidities such as coronary artery disease, arterial hypertension, and hypertensive heart disease [4]. Other common risk factors for developing CHF are being overweight or obese, type 2 diabetes mellitus, high blood cholesterol, metabolic syndrome, sleep apnea, smoking, and alcohol or drug abuse [5]. Increased mortality and poorer prognosis are expected with older age, male sex, low socioeconomic status, physical inactivity, and frequency of decompensations and hospitalizations [4]. Besides mortality, escalating health-care costs represent an increasing socioeconomic problem with a large part of the expenses being caused by hospitalization [2].

Damage of the heart and chronic pressure and/or volume overload triggers cellular, structural, and neurohumoral (mal-)adaptions [6]. This can lead to typical maladaptions and disease progression in CHF, and common symptoms like dyspnea, fatigue, decreased exercise, and physical capacity, swelling of lower extremities, and weight gain from fluid buildup, irrespectively of CHF classification (classified by preserved, moderate, or reduced systolic left ventricular ejection fraction) [4,6].

N-terminal natriuretic pro-B peptide (NT-proBNP) has emerged as an important diagnostic laboratory marker of cellular damage and CHF because blood concentrations increase as heart failure develops or worsens [4,6,7]. Accordingly, NT-proBNP is sensitive to early diagnosis of CHF or in the detection of deterioration during disease management [8].

The clinical severity of CHF is graded according to the New York Heart Association (NYHA) on the basis of clinical symptoms at various degrees of patients’ physical activity (PA) [9].

Therapy of CHF is primarily based on consistent drug intervention to reduce the cardiac workload and improve comorbidities [9]. Further therapy pillars include regular PA, weight and nutrition management, and patient self-management [9]. PA is an integral part of current guidelines for stable patients with CHF [10]. However, the level of global inactivity is pandemic; more than 25% of all adults fail to meet recommendations for PA set by WHO, with women being significantly less active [11,12]. Warburton et al. emphasize the paramount importance of PA, for the maximum physical performance represents the determining parameter for mortality, irrespectively of the stated disease [13]. In addition, deficiency symptoms, especially iron deficiency (ID), are known to occur in CHF Patients, as the ESC guidelines recommend iron therapy in case of ID in symptomatic CHF patients recently hospitalized for heart failure to reduce the risk of re-hospitalization [9].

A significant advantage of a specific diet for patients with CHF could not be proven so far, which is why the dietary recommendations for the general population of the German Society for Nutritional Medicine also apply to patients with CHF [4,14]. Although the beneficial properties of the MedDiet are associated with better health characteristics and better functioning, in particular with respect to coronary heart disease, there is little information on the effects of this diet on iron absorption, exercise capacity, and health-related quality of life (HrQoL) in CHF patients [15,16].

Exercise capacity in cardiopulmonary exercise tests correlates strongly with the degree of heart failure according to NYHA stages [17]. So far, it is unknown which other (medical) factors in CHF patients, like iron status or anemia, NT-proBNP value, anthropometric features, or a specific diet, such as the Mediterranean diet, have the greatest impact on exercise capacity. Vice versa, the current analyses will also investigate which of these parameters have an impact on iron status and anemia in patients with CHF.

## 2. Materials and Methods

### 2.1. Study Design

The objective of the presented data set refers to the baseline data from the “HITS” study (“**H**eart failure, **I**ndividual exercise training, **T**elemonitoring, **S**elfmanagement”) at the study center Hannover Medical School, Germany. HITS is a partially ongoing multicenter, prospective, both therapeutic and diagnostic, randomized, and parallel-group study done as a collaborative project between Leipzig University, Hannover Medical School, Leipzig Heart Center, AOKplus health insurances, IGES institute, Diaventions GmbH, and the Clinical Centers in Wolfsburg, Chemnitz and Dresden (Germany). The study is registered under DRKS00019022 in the German Clinical Trials Register. The institutional review boards of Hannover Medical School (No. 8786) and medical faculty of Leipzig University (479/19-ek) approved the study’s ethics, and written informed consent was obtained prior to the inclusion of study participants.

The HITS innovation fund project aims to establish a new care model that includes the early diagnosis of early stages of CHF, the treatment of CHF patients in accordance with the current ESC and AHA guidelines [9,18], avoidance of hospitalizations, and improvement in therapy adherence, exercise capacity, and HrQoL. 

### 2.2. Subjects

**Figure 1:**
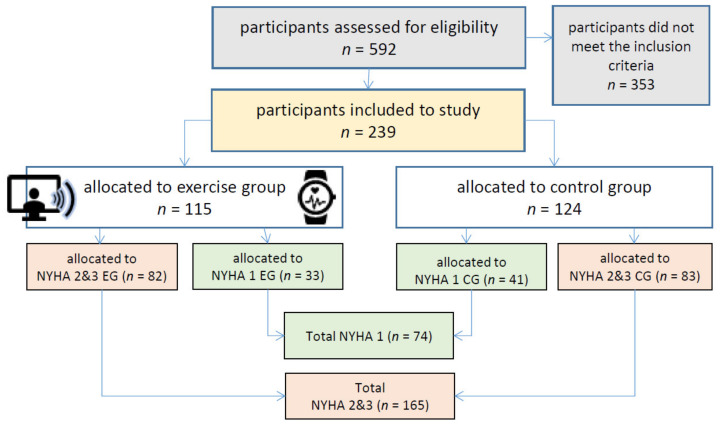
The course of study at baseline and randomization for the study center Hannover Medical School, Germany.

After several information events, newspaper articles, and online advertisements, a total of 592 subjects were assessed for eligibility at the study center Hannover Medical School, Germany, of which 353 subjects did not meet the inclusion criteria (see Figure 1).

According to predefined inclusion and exclusion criteria, we included female and male participants older than 18 years with diagnosed CHF. The NYHA functional classification was used to estimate patients’ functional ability based on their symptoms [17]. CHF Patients with NYHA classification I, II, and III, including cardiac support systems and transplants or implants were included in the study. We included both existing CHF patients and newly diagnosed patients. For patients with preserved ejection fraction and with risk factors for developing heart failure, a NT-proBNP value of over 125 pg/mL was set as the cutoff value for inclusion according to the ESC criteria [9]. Exclusion criteria were the lack of compliance, alcohol abuse or use of illegal drugs, active participation in other studies, and any physical or mental condition that precluded participation in an exercise intervention.

A total of 239 participants were randomly assigned to exercise (EG) and control group (CG). The allocation to the NYHA stages I, II, or III was taken from existing diagnoses or was determined by the study physicians based on the severity of symptoms during PA [9].

### 2.3. Procedures

Anthropometric data (body weight, height, waist, and hip circumference) were assessed based on defined standard operation procedures (SOP), after a general medical examination by a physician (including electrocardiogram, case history, and physical examination). Body-mass index (BMI) was calculated with the formula bodyweight (kg) ÷ height (m^2^). Fat-free mass and fat mass as markers of body composition were estimated by segmental, multifrequent, bioimpedance analysis (InBody720; Biospace, Seoul, Republic of Korea). In addition to cardiac-specific laboratory parameters [NT-proBNP, creatine kinase (CK), isoenzyme creatine kinase-MB (CK-MB), C-reactive protein (CRP), interleukine-6 (IL-6), and a safety blood profile including electrolytes, hemoglobin, hematocrit, thrombocytes, and leucocytes] which were collected via a venous blood sample, further laboratory parameters were collected at Hannover Medical School [ferritin (F), soluble transferrin receptor (sTfR), and transferrin saturation (TFS)].

When ferritin value was available (*n* = 216), participants were divided into one of the following three groups: ID according to ESC guidelines included participants (*n* = 82) with a ferritin level < 100 ng/mL or a ferritin level < 300 ng/mL in conjunction with a transferrin saturation < 20% [19]. Anemia included participants (*n* = 25) with a hemoglobin level < 12 g/dL for women and a hemoglobin level < 13 g/dL for men [19]. The group with normal iron status included all participants for which the two other criteria did not apply (*n* = 109). Additionally, we regarded sTfR values > 48.7 nmol/L as increased sTfR (*n* = 11). For this division, we did not include anemic participants.

Exercise capacity (measured as peak power output in watts [W]) and maximum heart rate (HR_max_) was assessed with an incremental exercise test on a bicycle ergometer (Ergoline P150, Bitz, Germany) until subjective exhaustion. The subjective perceived exertion was assessed by the Borg-Scale [20]. The exercise test started with a workload of 20 W or 50 W and was increased in 10-W or 17-W increments each minute. The test lasted until the participants were exhausted (60 revolutions per minute could not be maintained) or clinical findings for ergometry occurred (e.g., pathological blood pressure progression, pathological ECG changes). Additionally, a spirometric system (Oxycon CPX, CareFusion, Würzburg, Germany) was used to measure the oxygen uptake [VO_2_ (mL/min)], the carbon dioxide production [VCO_2_(mL/min)], and ventilation. All parameters were continuously measured breath-by-breath [21]. We recorded heart rate and blood pressure and collected arterial blood samples from the earlobe at rest, 1 min after the start, and every 3 min during the test to determine blood lactate concentrations (Ebio 6666, Eppendorf, Germany). The resting lactate + 1.5 mmol/L was set as the individual aerobic threshold. 

Among other questionnaires, we distributed a 14-item questionnaire as a primary measure to appraise adherence to the Mediterranean diet (MDS questionnaire) [22]. Scores ranged from 0 to 14 points, with higher scores indicating a higher adherence to the Mediterranean diet. A score of ≤5 points represents a low adherence, scores from 6–9 points represent a medium adherence, and ≥10 points represent a high adherence to MedDiet [22]. In order to be included in the overall evaluation, at least 12 of 14 questions had to be answered. Therefore, we assigned a score ≤5 points to low adherence, and a score ≥6 points to medium/high adherence to MedDiet.

The 23-item Kansas City Cardiomyopathy Questionnaire (KCCQ) was used to quantify physical limitations, symptoms, self-efficacy, social interference, and HrQoL in patients with CHF [23]. Scale scores are transformed to a 0 to 100 range; with lower scores representing lower levels of functioning. To facilitate interpretability, two summary scores were developed: functional status score and clinical summary score. A symptom scale, summarizing the results of the symptom frequency and symptom severity, was considered separately in this evaluation.

### 2.4. Statistical Analysis

First, the Kolmogorov-Smirnov test was used to test for normal distribution. Differences between the two groups were compared by Mann-Whitney-U-Test for non-parametric values, the Student *t*-test for unpaired samples, or the chi-square test. Parametric values were reported as mean and standard deviation (SD), non-parametric values were reported as median, minimum, and maximum values. For descriptive analysis, absolute frequencies were calculated for categorical variables, and mean and SD for continuous variables. Univariate associations between parameters were tested using Pearson’s correlation coefficient or non-parametric correlations for unpaired samples.

A stepwise backward multivariate linear regression was performed to identify parameters associated with relative oxygen uptake (VO_2max/kg_ [mL/min/kg]). A one-way analysis of variance (ANOVA) was used to test for group differences between normal iron status, ID, and anemia. The effect size is given by η². Significant differences were corrected with the Bonferoni post hoc test. The type-I-error was set to 5% (two-sided).

All statistical analyses were performed with IBM SPSS 28 Statistics (IBM Corporation, NY, USA). A priory case number calculation was performed to detect group differences of 10% in exercise capacity with the given number of cases with a statistical power of 0.85 and the mentioned significance.

## 3. Results

### Participants’ Characteristics

The combined data set consisted of 239 participants, 74 participants with NYHA 1 classification, and 165 participants with NYHA 2 & 3 classifications.

Table 1 shows subject characteristics for anthropometric and exercise-related parameters, iron-related laboratory parameters, as well as the results of the questionnaires KCCQ and MDS.

In addition to the primary diagnoses according to the inclusion criteria of the study, the most frequent secondary diagnoses are listed in Table 2 as relative indications.

In all participants, hemoglobin correlated with BMI (r = 0.183, *p* = 0.005), TSF% (r = 0.260, *p* < 0.001), KCCQ total score (r = 0.135, *p* = 0.046), ferritin (r = 0.170, *p* = 0.013), waist-to-hip-ratio (WHR [r = 0.323, *p* < 0.001]), watt_max_ (r = 0.279, *p* < 0.001), VO_2max_ (r = 0.337, *p* < 0.001), FFM (r = 0.368, *p* < 0.001), hematocrit (r = 0.956, *p* < 0.001), but not with sTFR (r = 0.039, *p* = 0.567). 

Ferritin correlates with BMI (r = 0.150, *p* = 0.003), FFM (r = 0.299, *p* < 0.001) and by trend with watt_max/kg_ (r = −0.124, *p* = 0.078) and VO_2max/kg_ (r = −0.124, *p* = 0.089). The sTFR correlated with BMI (r = 0.197, *p* = 0.003), KCCQ symptom scale (r = −0.209, *p* = 0.002), KCCQ functional score (r = −0.196, *p* = 0.004), clinical summery score (r = −0.200, *p* = 0.003) and KCCQ total score (r = −0.180, *p* = 0.008), watt_max/kg_ (r = −0.180, *p* = 0.009), and VO_2max/kg_ (r = −0.150, *p* = 0.035).

In a multivariate linear regression model including age, sex, WHR, NT-proBNP, ferritin, sTFR, tTSF(%), hemoglobin, hematocrit, VO_2max/kg_, individual aerobic lactate threshold, KCCQ, and MDS, it turned out that age (β = −0.230, *p* = 0.005), NYHA level (β = −0.225, *p* = 0.017), KCCQ symptom scale (β = 0.223, *p* = 0.017), individual aerobic lactate threshold (β = 0.184, *p* = 0.025), and MDS total score (β = 0.190, *p* = 0.020) predicted VO_2max/kg_. The model explained 31.1% of the total variation in VO_2max/kg_.

**Figure 2:**
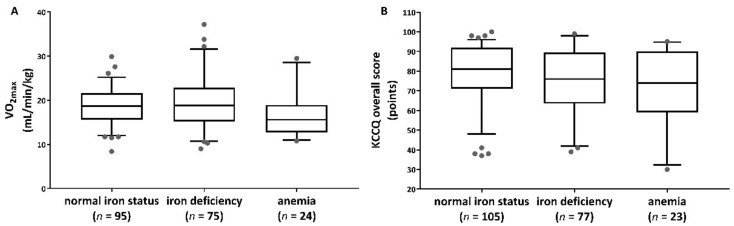
Group differences (normal iron status, iron deficiency, and anemia) related to relative oxygen uptake (**A**) and KCCQ overall score (**B**) by boxplots. Data that fall outside the Q1–Q3 range are shown as outliers of the data.

We divided the whole study group into three groups for normal iron status (*n* = 109), ID (*n* = 82), and anemia (*n* = 25). Table 3 shows the results of the ANOVA between the groups’ normal iron status, ID, and anemia. The three groups differed significantly in VO_2max_,VO_2max/kg_, KCCQ scores, and CRP (Table 3 and Figure 2).

A total of 154 participants completed the MDS questionnaire; 67 showed a low adherence to MedDiet and 87 showed a medium/high adherence. By this division, there are significant differences for body fat (*p* = 0.012) and VO_2max/kg_ (*p* = 0.043). The MDS correlated with BMI (r = −0.213, *p* = 0.006), body fat (r:−0.274, *p* = 0.002), watt_max/kg_ (r = 0.224, *p* = 0.004), VO_2max/kg_ (r = 0.243, *p* = 0.003), and WHR (r = −0.173, *p* = 0.029), but no correlation with the KCCQ subscores and the KCCQ overall score were found. 

In all, 62.1% of the participants with ID (*n* = 36) and 47.1% of the participants with anemia showed at least medium adherence to MedDiet. When comparing low adherence with medium/high adherence to MedDiet and ID and anemia classification, the chi^2^ test showed no significant differences (*p* = 0.476). 

Only 4.8% of all participants (*n* = 11) had increased sTFR. There were no significant differences between participants with increased sTFR and normal sTFR in physical performance, laboratory parameters, body composition, and MedDiet.

## 4. Discussion

We investigated the correlation between iron status, exercise capacity, and MedDiet dietary patterns in patients with CHF. 

According to guidelines, the treatment of CHF and associated comorbidities focuses on a combined strategy including a consistent drug intervention and a healthy lifestyle, including regular PA, and weight and nutrition management [9]. For the latter, we analyzed the adherence to MedDiet by questionnaire. The MedDiet includes higher consumption of olive oil, fresh fruits and vegetables, protein-rich legumes, fish, whole grains, and moderate amounts of wine and red meat [24]. Among several dietary patterns, the MedDiet is a well-known and well-studied healthy dietary pattern, and there is ample evidence supporting its efficiency to control cardiovascular risk factors, such as hypercholesterolemia, diabetes, and hypertension [25,26]. These findings could be primarily related to its anti-inflammatory and anti-oxidant properties as well as to the effectiveness of this dietary pattern in controlling waist circumference and obesity [27]. Yet, the results are conflicting and the discriminant factor to prescribe a MedDiet regime in CHF patients is represented by BMI [28].

The primary prevention for CHF is currently one of the main socioeconomic problems [29]. Avery et al. observed that CHF could be prevented with lifestyle changes, including changes in the individual dietary approach, like adherence to MedDiet [29]. Additionally, the results from an RCT in postmenopausal women combining MedDiet with exercise showed additional microvascular vasodilatory improvement, suggesting that the combination of exercise and dietary pattern in accordance with the MedDiet is an effective strategy to further reduce cardiovascular risk [30]. Our results are consistent with these findings and show a significant influence of MedDiet on VO_2max/kg_. VO_2max_ and relative VO_2max/kg_ is defined as the ability to transport and consume oxygen during exercise load and is related to cardiorespiratory fitness as a vital sign [31].

However, we could not find a significant correlation between MDS, ID, and anemia, even when comparing ID and anemia separately for low (≤5 points) and medium/high (≥6 points) adherence to MedDiet. This goes in line with the findings of a 12-month intervention study, in which, besides some positive effects on biomarkers of iron status, no overall effect on iron status was found [32]. With regard to the anti-inflammatory properties of the MedDiet, in particular the CRP value, we could not detect any correlation, contrary to previous study results [33,34]. When dividing the MDS score into low adherence and medium/high adherence to a MedDiet, there was still no difference between the two adherence groups in terms of the inflammatory marker CRP.

Tuttolomondo et al. describe that there has been no review of evidence base assessing the impact of dietary intake on functional indices that impact HrQoL in persons over 65 years of age [26]. To investigate this question for our patient collective, we examined the correlation between the KCCQ questionnaire and MDS overall score, as the KCCQ represents physical limitations, symptoms, self-efficacy, social interference, and HrQoL in patients with CHF [23]. However, we could neither find a correlation between high adherence to MedDiet and KCCQ subscores, nor the KCCQ symptom scale. In our evaluation of CHF patients, no indications could be found in this regard either. With the aging of society, there is concern that people may be living longer with a disability or impaired quality of life [24]. The question of which nutritional factors are suitable to improve HrQoL has yet to be answered.

Iron deficiency is prevalent in up to 50% of patients with CHF, which is associated with reduced exercise capacity, impaired HrQoL, and increased risk of hospitalization and mortality, regardless of the presence or absence of anemia [35,36]. This includes that an acute reduction of blood hemoglobin concentration (Hb) results in lower VO_2max_ and endurance performance, due to the reduction of the oxygen-carrying capacity of blood [37].

Indeed, our analyses showed significant differences between anemic and non-anemic participants in terms of VO_2max_ and significant group differences between the three iron status groups (normal iron status, ID, and anemia) in terms of VO_2max_ and VO_2max/kg_ and KCCQ questionnaire as an expression of functional outcomes.

The presence of ID or anemia are frequent co-occurrences in patients with CHF whereby an absolute ID with depleted iron stores can be distinguished from a functional ID caused by impaired iron utilization or misdistribution [38,39]. That is in line with our results showing that a total of 48.9% of our participants had ID or anemia.

The current definition of ID for CHF patients is a ferritin level < 100 ng/mL, or ferritin levels from 100–299 ng/mL in the presence of TSF level < 20%, irrespectively of present anemia [19]. These high values were set arbitrarily considering that ferritin is an acute phase protein and levels are increased in chronic disease and inflammation [39]. Referring to this, 37.9% of our patients were found to have an ID. Several studies including meta-analyses found improvements following intravenous iron substitution with ferric carboxymaltose or iron sucrose, not only in cardio-specific questionnaires, but also in regard to all cardiovascular deaths and hospitalization [19,40,41,42,43]. Therefore, the ESC guidelines recommend early diagnosis and treatment of ID in patients with CHF [9]. Not least because of increased healthcare costs in case of untreated ID [44].

An alternative to ferritin determination could be the combined evaluation of low hepcidin and increased sTfR values. The sTfR value is expressed more abundantly in intracellular ID and indicates increased tissue demand even before low hemoglobin levels occur. Unlike ferritin and transferrin, it is not affected by inflammation or vigorous physical exercise [45]. Jankowska et al. calculated a high predictive value for all-cause mortality within 12 months, when considering both sTfR and hepcidin levels. The combination of these two values proposed a new definition ID [46]. Currently, this is not yet used in clinical routines due to difficulties in hepcidin determination. We did not collect hepcidin but found 4.8% of our patients with increased sTfR according to the standardized laboratory values of Hannover Medical School.

### Strengths and Limitations

Our study has strengths and limitations. One limitation of our multicenter study that we have to state is that the technologies used to determine the body compositions, the exercise capacity, and the consulted laboratories were not the same for all study centers. In addition, not every parameter on which this analysis is based has been collected in every study center, which is why the present analysis included only participants of Hannover Medical School (*n* = 239) instead of the whole study population (*n* = 679), which would have resulted in a greater statistical power. Overall, not all of the study data collected, including medications or nutritional supplement, is yet available, as the examinations are still ongoing in some study centers.

For the diagnosis of ID, the current diagnostic criteria for clinical heart failure were also applied to NYHA 1 patients. Since these were often just patients at risk for developing heart failure, too many patients may have been diagnosed with ID, which could also explain the relatively good exercise performance in the group of patients with ID.

## 5. Conclusions

Our results show that anemia in patients with CHF has an impact on both maximum oxygen uptake as a vital sign for cardiorespiratory fitness, and functional outcomes by questionnaire. Furthermore, our results show the higher a person’s adherence to MedDiet, the higher the relative cardiorespiratory fitness. The analysis of our baseline data show, that 10.9% of study participants are anemic and 37.9% showed an ID. In further studies, we suggest investigating whether or not the combined evaluation of low hepcidin and increased sTfR values could be an alternative to ferritin determination.

Only 7.8% have stated a high adherence to the MedDiet. Both symptomatic and non-symptomatic study participants failed to meet reference values for physical performance during exercise testing [47]. With our analysis, we were able to identify gaps in iron supply, but also with regard to healthy nutrition and PA, so we encourage enforcing the guidelines in the diagnosis and treatment of CHF more strongly.

## Figures and Tables

**Table 1:** Subject characteristics.

	NYHA 1	*n*	NYHA 2&3	*n*	*p*-Value
Sex (women/men)	26/48	74	74/91	165	0.159
Age (years)	65 (36;81)	74	68 (27;94)	165	0.020
Body weight (kg)	79.2 (53.3;126.0)	74	82.7 (45.3;153.0)	165	0.175
Body mass index [BMI (kg/m^2^)]	25.2 (19.1;42.1)	74	28.2 (17.2;52.3)	165	0.001
Body fat [FM (kg)]	23.3 (6.1;53.8)	53	27.7 (6.1;88.8)	123	0.004
Fat free mass [FFM (kg)]	55.3 (41.1;79.5)	53	55.4 (33.7;84.6)	123	0.757
Waist circumference (cm)	96 ± 14	73	100 ± 14	160	0.019
Hip circumference (cm)	101 (85;129)	70	105 (77;150)	152	0.005
Waist-to-hip-ratio	0.93 ± 0.08	70	0.95 ± 0.09	152	0.212
Exercise capacity (watt_max_)	130 (50;267)	72	110 (50;217)	147	<0.001
Relative exercise capacity (watt_max/kg_)	1.71 (0.67;2.98)	72	1.28 (0.54;2.60)	147	<0.001
Relative oxygen uptake [VO_2max/kg_ (mL/min/kg)]	21.2 (8.4;37.2)	68	16.3 (9.0;29.5)	138	<0.001
Individual aerobic lactate threshold [base lactate + 1.5 mmol/L (mmol/L)]	2.57 (2.00;4.64)	71	2.50 (2.00;5.42)	147	0.359
Ferritin [F(ng/mL)]	129 (29;466)	68	125 (10;1269)	143	0.779
CRP (mg/L)	1.0 (0.6;9.5)	72	1.3 (0.5;43.2)	160	0.047
Soluble transferrin receptor [sTfR(nmol/L)]	30.4 (8.7;52.8)	72	32.6 (12.8;115.0)	156	0.042
Transferrin saturation [TSF(%)]	32 (16;62)	73	29 (8;81)	161	0.020
Hemoglobin (g/dL)	14.3 (11.3;17.3)	72	13.9 (10.4;19.8)	157	0.030
Hematocrit (L/L)	0.42 ± 0.04	72	0.41 ± 0.04	150	0.046
NT-proBNP (ng/L)	237 (50;1882)	74	320 (36;1848)	164	0.003
KCCQ total score	90.0 (59;100)	68	73.0 (30;98)	159	<0.001
KCCQ functional status score	93.8 (65.4;100.0)	68	78.6 (25.0;100.0)	159	<0.001
KCCQ clinical summery score	94.0 (57.5;100.0)	68	73.8 (25.0;100.0)	159	<0.001
KCCQ symptom scale	96.9 (57.1;112.5)	68	81.3 (21.9;100.0)	159	<0.001
MDS total score	6.0 (1;13)	48	6.0 (0;13)	118	0.331

Parametric values were reported as mean and SD, non-parametric values were reported as median, min, and max values. KCCQ = Kansas City Cardiomyopathy Questionnaire; MDS = Mediterranean Diet Score.

**Table 2:** Overview of diagnoses as relative values in percent.

Secondary Diagnoses	%
Coronary artery disease	73.2
Hypertension	68.7
Hypercholesterolemia	51.5
Atrial fibrillation and other cardiac arrhythmia	47.0
Vitium cordis	38.9
History of cardiac support systems; transplants or implants	38.4
Chronic renal failure	25.8
Orthopedic impairment	20.2
Other diseases	19.7
Diabetes mellitus	18.2
Obesity	13.1
Other atherosclerotic diseases	12.6
Left bundle branch block	6.6
Malignoma	5.6
Thyroid disorder	5.6
Dilated cardiomyopathy	5.1
Chronic obstructive pulmonary disease and bronchial asthma	5.1
Pulmonary hypertension	3.5
Right bundle branch block	3.5
Neurological and psychiatric disorders	3.5
Hypertensive heart disease	3.0
Other cardiomyopathies	3.0

**Table 3:** ANOVA Results of the subgroups for iron status.

	Normal Iron Status	*n*	Iron Deficiency	*n*	Anemia	*n*	*p*-Value	η*²*
Age (years)	68 (30;83]	109	65 (27;81)	82	71 (34;94)	25	0.309	0.01
Weight (kg)	87.8 (55.1;153.0)	109	75.5 (53.2;120.2)	82	72.3 (55.2;94.4)	25	0.012	0.04
Body mass index [BMI(kg/m^2^)]	27.7 (19.2;52.3)	109	27.3 (17.2;38.2)	82	27.6 (19.0;46.6)	25	0.173	0.02
Body fat (kg)	26.3 (8.9;88.8)	81	27.6 (6.1;50.3)	64	22.4 (7.8;52.4)	16	0.536	<0.01
Fat free mass [FFM(kg)]	56.4 (33.7;79.5) ^a^	81	50.4 (36.8;84.6) ^a^	64	58.4 (37.6;71.3)	16	0.010	0.06
Waist-to-hip-ratio (WHR)	0.96 (0.75;1.15)	102	0.92 (0.78;1.07)	80	0.95 (0.69;1.10)	23	0.035	0.03
Exercise capacity (watt_max_)	120 (50;250) ^b^	104	110 (50;267)	78	100 (50;160) ^b^	25	0.049	0.03
Relative exercise capacity (watt/kg)	1.45 (0.67;2.68)	104	1.49 (0.54;2.98) ^c^	78	1.11 (0.57;2.60) ^c^	25	0.032	0.03
Oxygen uptake [VO_2max_ (mL/min)]	1502 (761;2797) ^b^	95	1392 (721;3330)	75	1351 (785;1881) ^b^	24	0.021	0.04
Relative oxygen uptake [VO_2max/kg_ (mL/min/kg)]	18.55 ± 4.05	95	19.39 ± 5.86 ^c^	75	16.52 ± 4.80 ^c^	24	0.046	0.03
Individual aerobic lactate threshold [base lactate + 1.5 mmol/L (mmol/L)]	2.55 (2.00;5.42)	104	2.49 (2.00;3.98)	76	2.55 (2.06;3.39)	24	0.494	<0.01
CRP (mg/L)	1.2 (0.5;23.7) ^b, c^	109	1.0 (0.6;21.9) ^c^	82	2.9 (0.6;43.2) ^b, c^	24	0.002	0.06
NT-proBNP (ng/L)	283 (50;1335)	109	251 (36;1882)	82	335 (127;1304)	25	0.951	<0.01
KCCQ total score	81 (37;100)	105	76 (39;99)	77	74 (30;95)	23	0.050	0.04
KCCQ functional status score	88 (34;100)	104	80 (32;100)	77	82 (25;100)	25	0.040	0.03
KCCQ clinical summery score	85 (36;100)	104	79 (37;100)	77	74 (25;100)	25	0.038	0.03
KCCQ symptom scale	89 (34;100)	104	84 (28;113)	77	78 (22;100)	25	0.022	0.04
MDS total score	6 (2;13)	79	6 (1;13)	58	5 (0;10)	17	0.702	<0.01

Parametric values were reported as mean and SD, non-parametric values were reported as median, min, and max values. η²: effect size eta squared. ^a^: Post hoc shows significant differences between groups with normal iron status and iron deficiency (ID). ^b^: Post hoc shows significant differences between groups with normal iron status and anemia. ^c^: Post hoc shows significant differences between groups ID and anemia.

## Data Availability

The datasets used and/or analyzed during the current study are available from the corresponding author upon reasonable request.
